# The National Non-deanery Training Initiative: A National Needs Assessment and Roadmap for Improving the Training of Trauma and Orthopaedic Non-deanery Trainees

**DOI:** 10.7759/cureus.112315

**Published:** 2026-07-09

**Authors:** Mohamed Hashem, Hatem Hussein

**Affiliations:** 1 Orthopaedics, Royal Blackburn Teaching Hospital, London, GBR; 2 Trauma and Orthopaedics, Southend University Hospital, Essex, GBR

**Keywords:** cesr, curriculum, education, img, nhs workforce, portfolio pathway, sas, trauma & orthopaedics

## Abstract

The UK trauma and orthopaedic (T&O) workforce increasingly relies on non-deanery doctors, including international medical graduates (IMGs), Specialist and Associate Specialist (SAS) doctors, and locally employed doctors, who contribute substantially to service delivery across elective and trauma care. Despite their important role, there remains no nationally standardised training pathway to support competency development and progression through the Portfolio Pathway towards specialist registration. This technical report proposes a structured, curriculum-aligned framework for non-deanery T&O registrars that integrates educational supervision, workplace-based assessments, mentorship, operative competency monitoring, portfolio development, leadership activities, and protected educational time within existing NHS infrastructure. The proposed model aims to convert routine clinical service into documented educational progression while providing a framework that may support greater consistency in training, improve educational equity, and strengthen workforce retention. Further evaluation is required to determine its impact on educational and patient outcomes. A phased implementation strategy incorporating national governance, local supervision, and quality assurance mechanisms is described. Adoption of such a framework could provide a practical and scalable solution to support consultant-level competency development and strengthen the long-term sustainability of the UK T&O workforce.

## Introduction

The UK National Health Service (NHS) trauma and orthopaedic (T&O) workforce is increasingly reliant on doctors working outside nationally recognised postgraduate training programmes, including Locally Employed Doctors (LEDs), International Medical Graduates (IMGs), Medical Training Initiative (MTI) doctors, clinical fellows, and Specialty and Associate Specialist (SAS) doctors [1].

Collectively, these doctors make a substantial contribution to elective and trauma services, frequently undertaking frontline clinical responsibilities that are essential to maintaining service delivery in the face of growing workforce shortages and increasing patient demand.

In this article, the term non-deanery doctors refers to T&O doctors working outside a nationally recognised UK postgraduate training programme, irrespective of their contractual grade or country of primary medical qualification. Although these clinicians represent a heterogeneous workforce with differing educational backgrounds, contractual arrangements, and career aspirations, they share the common challenge of pursuing professional development without the structured educational governance routinely available within recognised training programmes. The importance of this workforce continues to grow. The General Medical Council (GMC) Workforce Report 2024 identified 87,151 licensed doctors across the UK who were neither enrolled in postgraduate training nor included on the GP or Specialist Register, including 66,292 doctors in England and Wales, making this the fastest-growing segment of the UK medical workforce.

For readers unfamiliar with the UK postgraduate training system, doctors pursuing a career in trauma and orthopaedic surgery normally enter a nationally recognised speciality training programme through a competitive selection process. Progression is competency-based and aligned with the 2021 Trauma and Orthopaedic Curriculum, supported by structured educational supervision, workplace-based assessments, Annual Review of Competence Progression (ARCP), and successful completion of training leading to the award of a Certificate of Completion of Training (CCT). However, progression is not always linear, and many trainees require additional training time because of competency acquisition, research, parental leave, changes in curriculum requirements, or other personal and professional circumstances. Doctors working outside recognised training programmes generally progress towards specialist registration through the GMC Portfolio Pathway (formerly the Certificate of Eligibility for Specialist Registration (CESR)), demonstrating equivalence to the CCT through comprehensive evidence mapped against the T&O curriculum and GMC standards [1,2].

The Portfolio Pathway represents an important and well-established route to specialist registration for doctors who have not completed a UK-approved training programme [1,2]. Successful applications require comprehensive evidence across all domains of the T&O curriculum, including operative competence, clinical decision-making, leadership, governance, education, research, and professional development, with documentation mapped to the Intercollegiate Surgical Curriculum Programme (ISCP) [2]. Without a structured educational framework embedded in routine clinical practice, evidence generation frequently becomes retrospective, fragmented, and highly dependent on local educational support, often prolonging progression despite appropriate clinical experience.

Current challenges include geographical and organisational variation in training opportunities, inconsistent quality and continuity of educational supervision, variable operative exposure, and the considerable administrative burden associated with portfolio development. Importantly, these challenges are not unique to non-deanery doctors; trainees within recognised postgraduate programmes may also experience delays in competency acquisition and progression because of service pressures or limited training opportunities. However, non-deanery doctors often encounter these challenges without the continuity of educational governance, curriculum oversight, and structured progression processes available within formal training programmes [3-5]. Ensuring equitable access to high-quality education and robust competency assessment for this expanding workforce is therefore both a workforce priority and an important component of maintaining high standards of patient care [5,6].

Recognising this need, we established the National Non-deanery Training Initiative, a national educational initiative that seeks to identify the educational needs of non-deanery T&O doctors and propose a nationally coordinated training framework aligned with the UK T&O curriculum. Rather than creating a parallel training pathway, the initiative aims to build upon existing NHS educational infrastructure, local educational programmes, and professional body resources to provide a more consistent, equitable, and transparent approach to competency development, portfolio progression, and career advancement. By supporting educational quality and workforce development, the framework has the potential to improve training consistency, enhance workforce retention, and contribute to the long-term sustainability of the T&O workforce. Its educational, workforce, and patient-level impact, however, will require prospective evaluation.

The proposed framework is grounded in the principles of competency-based medical education (CBME), in which progression is determined by the demonstration of competence rather than time served. It incorporates established educational strategies, including structured educational supervision, workplace-based assessment, reflective practice, and portfolio-based assessment, which underpin the UK T&O curriculum [4,7,8]. The framework also draws upon experiential learning theory, recognising that competence develops through supervised clinical experience, feedback, reflection, and deliberate practice, while structured mentorship supports professional development and career progression [8]. By integrating these established educational principles within existing NHS structures, the framework provides an evidence-informed approach to supporting doctors working outside nationally recognised postgraduate training programmes.

This study represents the first phase of the National Non-deanery Training Initiative. It presents a conceptual, evidence-informed framework intended to stimulate national discussion and inform stakeholder engagement, consensus development, pilot implementation, and formal evaluation before wider adoption.

## Technical report

Background/rationale

The UK relies increasingly on experienced, internationally trained surgeons entering the NHS outside formal training programmes. Many of these colleagues have completed recognised training abroad and arrive with senior clinical skills, yet they often struggle to demonstrate the breadth of competencies aligned with UK curriculum standards. This creates a gap between their real-world capability and the level of documented evidence required for specialist recognition.

Under the current Portfolio Pathway, doctors must evidence consultant-level competence across all surgical domains: operative proficiency, decision-making, leadership, education, audit, research, and professional development [6]. Similar challenges have been recognised across surgical specialities and have prompted calls for improved support structures for SAS and locally employed doctors [7]. However, many non-deanery registrars are unfamiliar with the UK's documentation-driven training culture and do not fully recognise the importance of structured portfolio progression. They also report challenges related to navigating NHS training structures, educational supervision, and career progression pathways. Moreover, many do not fully appreciate these requirements until later in their career progression, often after success in the FRCS (Fellowship of the Royal Colleges of Surgeons) examination. By this stage, retrospective evidence gathering becomes difficult, frustrating, and expensive, sometimes leading to failure or abandonment of the process altogether.

Additional barriers include limited early guidance regarding curriculum alignment, inconsistent opportunities to obtain appropriate operative sign-off evidence, insufficient protected supporting professional activity (SPA) time for portfolio development, variable access to supervisors familiar with workplace-based assessments, and a predominantly reactive approach to evidence collection. Consequently, many doctors experience delays in progression despite acquiring the clinical competencies expected of future consultants.

This results in prolonged progression timelines, reduced morale, and the loss of talented surgeons who could otherwise progress to consultant roles, impacting workforce sustainability and patient care.

Yet, these doctors already contribute a significant share of service delivery. They attend theatres, fracture clinics, MDTs, governance meetings, and on-call rotas. The NHS already provides them with the clinical exposure and opportunities needed to reach consultant standard, but without a structured framework, much of that learning is unrecorded, unrecognised, and wasted from a training perspective.

Introducing a clear, nationally recognised, competency-mapped training pathway would allow the system to directly convert existing service activity into structured training outcomes, ensure continuous evidence generation throughout development, support FRCS examination readiness and post-exam portfolio completion, and achieve consultant-level capability with greater efficiency, consistency, and safety.

This represents a cost-effective workforce development intervention: better-trained surgeons, clearer progression pathways, and improved retention, leading to safer patient care.

By providing equitable access to existing educational resources, including outpatient clinics, operating lists, clinical supervision, and quality improvement opportunities, a structured national training framework could help non-deanery doctors acquire the competencies expected of nationally recognised training programmes. Such an approach has the potential to support career progression, improve educational consistency, and enhance trainee experience. Further evaluation will be required to determine its impact on educational and workforce outcomes.

A stronger, structured national framework is not a luxury, but an increasingly important workforce priority.

Proposed pathway structure

This proposal seeks to introduce a practical and scalable national framework that leverages existing NHS training and educational structures. This framework is intended to build upon existing educational infrastructure rather than create a parallel training system. While implementation would require investment in supervision, governance, and faculty development, these costs are likely to be offset by improvements in workforce development, retention, and training efficiency. Formal economic evaluation is required.

The pathway is designed to ensure curriculum-aligned competency progression, continuous evidence generation, and transparent oversight throughout the development of non-deanery T&O registrars.

The proposed national training framework consists of several interdependent components designed to facilitate curriculum-aligned progression, competency acquisition, and portfolio development. These core elements and their implementation strategies are summarised in Table 1. 

**Table 1:** Core components of the pathway T&O: trauma and orthopaedics, CS: Clinical Supervisor, ES: Educational Supervisor, WBAs: workplace-based assessments, SAS: Specialty and Associate Specialist, FRCS: Fellowship of the Royal Colleges of Surgeons, IMG: international medical graduate, PBAs: Procedure-Based Assessments, CBDs: Case-Based Discussions, DOPS: Direct Observation of Procedural Skills, QI: quality improvement, M&M: morbidity and mortality, SPA: supporting professional activities, NHS: National Health Service, BOA: British Orthopaedic Association.

Component	Description and Implementation Strategy
Curriculum-aligned training	Learning plans mapped directly to the T&O curriculum, incorporating milestone-based progression and targeted skill acquisition.
Formal supervision structure	Each registrar is assigned both a Clinical Supervisor (CS) and Educational Supervisor (ES), with structured learning reviews conducted at least monthly to monitor progression and ensure WBAs and operative experience align with identified goals.
Structured mentorship	Access to a national SAS mentorship network providing career navigation, FRCS guidance, and IMG pastoral support to reduce isolation and enhance integration.
Workplace-Based Assessments (WBAs)	Regular PBAs, CBDs, DOPS and reflective assessments embedded within routine clinical activities, mapped to the relevant sections of the curriculum and supervised by trained assessors.
Operative logbook and consolidation	Quarterly evaluation of case volume, case mix and index procedures, with targeted action planning to ensure breadth aligned with consultant-level competence expectations.
Academic, research and teaching engagement	Mandatory participation in departmental audit, QI, journal clubs and undergraduate/postgraduate teaching, with documented roles and outputs uploaded to the e-portfolio.
Leadership and governance	Exposure to M&M review, patient safety audits, guideline development and clinical meetings, supporting development of non-technical and regulatory competencies.
Protected SPA time	Job-planned, contractual allocation of SPA sessions in line with NHS England SAS development guidance, ensuring time for portfolio completion, reflection, and exam preparation.
E-Portfolio standardisation	Utilisation of a nationally consistent portfolio template aligned with Portfolio Pathway evidence requirements to ensure efficient, timely documentation and simplified signposting.
National quality assurance and benchmarking	Oversight from BOA and SAS educational governance structures to promote equity, consistency, and measurable standards across participating Trusts.

Effective educational supervision is a recognised determinant of successful competency progression and educational outcomes [8]. Although some NHS organisations allocate educational or clinical supervisors to non-deanery doctors, the structure, consistency, and protected time available for supervision vary considerably, resulting in differences in educational support and career progression. Workplace-based assessments are widely accepted as valid educational tools that support feedback, progression, and competency development [9,10].

International medical graduates frequently report challenges related to supervision and career progression [11,12]. This structured approach ensures that existing service delivery is converted into documented training progress, rather than relying on retrospective evidence generation. Protected training time is emphasised as an investment in patient safety and professional development, not an optional luxury.

By creating a clearly defined, curriculum-driven training pathway, non-deanery registrars can achieve competency progression comparable to that of colleagues in national training posts, supporting safe and confident transition to independent consultant practice. Formal mentorship programmes have been associated with improved professional development, career satisfaction, and career progression [13].

Implementation strategy 

Implementation of the proposed pathway would require a phased national approach involving governance development, pilot testing, evaluation, and subsequent wider adoption. The proposed implementation roadmap is outlined in Table 2.

**Table 2:** Proposed implementation roadmap for a national non-deanery T&O training pathway BOA: British Orthopaedic Association; SOP: standard operating procedure.

Phase	Timeline	Expected Output
National Working Group Formation	Months 0-3	Governance structure established and deliverables agreed
Pilot Programme Design	Months 3-6	Standard operating procedures, documentation templates, and evaluation framework developed
Pilot Launch	Month 6	Participating registrars enrolled and supervisors trained
Evaluation Phase	Months 12-18	Outcome data collected and pathway refined
National Roll-Out	Year 2 onwards	National implementation with BOA endorsement

Before national implementation, the proposed framework should undergo prospective pilot evaluation (Table 3) across a small number of NHS trauma and orthopaedic departments representing different organisational settings. The pilot evaluation should assess feasibility, acceptability, educational effectiveness, implementation fidelity, and resource requirements. Outcomes could include trainee engagement, workplace-based assessment completion, operative exposure, portfolio progression, educational supervision quality, trainer and trainee satisfaction, progression towards the Portfolio Pathway, and workforce retention. Findings from the pilot would inform refinement of the framework before wider regional and national implementation.

**Table 3:** Suggested pilot design NHS: National Health Service, T&O: trauma and orthopaedics, WBAs: workplace-based assessments, SPA: supporting professional activity.

Component	Proposal
Design	Prospective multicentre pilot
Sites	5-10 NHS T&O departments
Duration	12 months
Participants	Non-deanery T&O doctors
Comparator	Standard local training
Primary outcome	Feasibility and implementation
Secondary outcomes	WBAs, operative numbers, portfolio progress, supervision quality, satisfaction, retention
Economic outcomes	SPA time, faculty time, administration costs, cost per trainee
Evaluation	Mixed-methods (quantitative + qualitative interviews)

Alongside the pilot evaluation, a formal health economic evaluation should be undertaken to quantify implementation costs, including educational supervisor SPA time, faculty development, administrative support, quality assurance, and governance. These costs should be considered alongside potential benefits, including improved workforce retention, reduced recruitment costs, enhanced educational efficiency, and accelerated progression towards specialist registration. Such an evaluation would provide commissioners with evidence regarding the cost-effectiveness and sustainability of the framework.

Challenges and mitigation strategies

While the proposed pathway is achievable using current infrastructure, several important challenges must be acknowledged and proactively addressed to ensure successful national implementation. The anticipated implementation challenges and proposed mitigation strategies are summarised in Table 4. 

**Table 4:** Challenges and mitigations strategies CCT: Certificate of Completion of Training, CESR: Certificate of Eligibility for Specialist Registration, GMC: General Medical Council, ISCP: Intercollegiate Surgical Curriculum Programme, JCST: Joint Committee on Surgical Training, NHS: National Health Service, SAS: Specialty and Associate Specialist, SPA: supporting professional activities, BOA: British Orthopaedic Association, SOPs: standard operating procedures, WBA: workplace-based assessment, ARCP: Annual Review of Competence Progression, RCS England: Royal College of Surgeons of England, FRCS: Fellowship of the Royal Colleges of Surgeons.

Challenge	Mitigation Strategy
Short-term employment contracts for non-deanery registrars	Advocate for minimum 3-year developmental contracts to allow sufficient time for curriculum-aligned progression, FRCS preparation, and portfolio evidence generation. Alignment with BOA workforce guidance and local SAS Tutors’ business cases will strengthen engagement from Trust leadership and Medical Directors.
Service pressures limiting access to structured training	Embed protected SPA and training time within job plans as a patient safety imperative, supported by national BOA and NHS England SAS Development Guidance. Workforce planning should reinforce that training enhances long-term service delivery capacity.
Variable educational culture between hospital sites	Introduce national benchmarking, shared outcome dashboards, and inter-site peer review to support consistency. Clear expectations for supervisors and mentorship roles should be operationalised through BOA-supported SOPs.
Need for supervisor training and portfolio literacy	Expand BOA and RCS England SAS educational faculty development programmes to ensure supervisors are skilled in WBA completion, curriculum signposting, and CESR/Portfolio Pathway requirements.
Local governance and faculty structure not yet formalised	Develop local ARCP-style panels comprising educational supervisors and approved faculty, supported through BOA guidance. National oversight ensures consistency and avoids undue local variation in progression decisions.
Stakeholder approval and alignment	Secure endorsement from BOA, JCST, NHS England and ISCP to ensure consistency with existing curricula and evidence standards. Early collaboration reduces implementation friction and enhances legitimacy of outcomes.
Mindset and perception differences between CCT and Portfolio Pathway graduates	Promote evidence-based equivalence policies, educational sessions, and consultant-led advocacy to challenge outdated stigma and reinforce that both pathways achieve the same GMC specialist registration standards.

Broader workforce considerations

Implementing this pathway requires a clear cultural shift. Non-deanery registrars must be recognised not solely as service providers, but as future consultants who deserve fair and structured developmental opportunities. Successful implementation will require robust national governance, transparent competency standards, educationally engaged supervisors, and consistent adoption across participating organisations. When these elements are established, the proposed pathway offers a sustainable mechanism for supporting workforce development while promoting educational equity and high standards of patient care.

With these enablers in place, the proposed pathway offers a sustainable solution to a well-recognised workforce challenge while promoting equity, morale, and patient-centred outcomes.

## Discussion

This study presents a proposed national educational framework to support the training and career progression of non-deanery T&O doctors. Rather than creating a parallel training pathway, the framework seeks to coordinate existing educational opportunities within a structured, curriculum-aligned model that facilitates competency development, prospective portfolio evidence generation, and progression through the GMC Portfolio Pathway [1,2,4,6]. By integrating established educational processes within routine clinical practice, the framework addresses variation in educational support for doctors working outside nationally recognised postgraduate training programmes while building upon existing NHS educational structures.

The proposal is particularly relevant given the increasing reliance of the NHS on non-deanery doctors to sustain trauma and orthopaedic services. This workforce includes locally employed doctors, IMGs, MTI doctors, speciality doctors, and clinicians pursuing the Portfolio Pathway, all of whom make substantial contributions to service delivery [5,14,15]. However, non-deanery status and IMG status are not synonymous. Many UK graduates work in non-deanery posts, while many IMGs successfully progress through recognised UK training programmes. Furthermore, doctors entering UK practice from overseas may initially face additional challenges related to adapting to NHS systems, professional culture, and clinical governance that differ from those experienced by UK-trained locally employed doctors [11,12]. National workforce reports continue to demonstrate disparities in access to postgraduate training, educational opportunities, and career progression, particularly among IMGs, despite their increasing contribution to the NHS workforce [5,14]. These findings support the need for educational models that are sufficiently flexible to address the differing needs of these groups while maintaining consistent national standards.

Importantly, the proposed framework is intended to complement rather than replace the existing Portfolio Pathway. The Portfolio Pathway remains a robust and well-established route to specialist registration for doctors who have not completed a UK-approved training programme [1,2,6]. Successful applications require comprehensive evidence across all domains of the T&O curriculum, including operative competence, clinical judgement, leadership, governance, teaching, research, and professional development [2,4,6]. Although the pathway itself is well established, evidence generation frequently remains retrospective, fragmented, and dependent upon local educational support. Embedding curriculum mapping, structured educational supervision, and prospective portfolio development within routine clinical practice may therefore improve consistency and transparency while supporting more efficient progression without altering the standards required for specialist registration.

The framework should also be viewed alongside existing educational initiatives supporting doctors outside recognised training programmes. Non-training doctors may access the Intercollegiate Surgical Curriculum Programme (ISCP) ePortfolio to facilitate curriculum mapping and competency recording [4]. Similarly, the Royal College of Surgeons of England SAS Strategy advocates improved educational opportunities, career development, and equitable access to training for SAS and locally employed doctors [7]. Furthermore, the 2021 Trauma and Orthopaedic Curriculum, with its emphasis on competency-based progression and Capabilities in Practice (CiPs), provides an educational framework that aligns closely with the principles underpinning this proposal [4]. Several NHS organisations have also developed local educational initiatives supporting doctors pursuing the Portfolio Pathway, although provision remains variable across the UK. Rather than replacing these existing initiatives, the National Non-deanery Training Initiative seeks to provide a nationally coordinated framework that builds upon examples of good practice, reduces geographical variation, and promotes greater educational consistency while preserving flexibility for local implementation.

The educational principles underpinning the framework are well established. Competency-based medical education emphasises progression through the demonstration of competence rather than time served, with educational supervision, workplace-based assessment, and reflective practice forming the cornerstone of contemporary postgraduate surgical training [8-10]. Workplace-based assessments are most effective when embedded within a longitudinal developmental process that provides regular feedback and supports progressive competency acquisition rather than functioning solely as summative assessments [9,10]. Similarly, structured mentorship has been associated with improved professional development, career satisfaction, and career progression among healthcare professionals [13]. These benefits may be particularly valuable for doctors unfamiliar with UK training structures or navigating alternative routes to specialist registration [11-13]. Integrating these established educational principles within a coherent national framework, therefore, provides an evidence-informed approach to supporting doctors working outside recognised postgraduate training programmes.

A major strength of the proposed framework is that it builds upon educational infrastructure already available within NHS organisations. Outpatient clinics, operating theatres, trauma meetings, multidisciplinary team discussions, governance activities, and workplace-based assessments already provide valuable opportunities for competency development. The framework seeks to organise these existing educational resources within a structured, curriculum-aligned model that promotes continuous competency development, prospective portfolio evidence generation, and educational quality assurance. Importantly, implementation is not without resource implications. Dedicated SPA time for educational supervisors, faculty development, administrative support, and local governance will be required and should be recognised within consultant job planning and workforce planning. Although the framework is designed to complement existing educational programmes rather than establish a parallel system, a formal health economic evaluation will be essential to determine implementation costs, cost-effectiveness, and longer-term workforce benefits.

Successful implementation will require collaboration between NHS organisations, educational supervisors, professional bodies, and national stakeholders. We recognise that postgraduate surgical education is currently delivered within an increasingly pressurised healthcare system where consultant supervisory capacity, service demands, and workforce shortages already present significant challenges. Furthermore, many organisations already provide excellent educational support for non-deanery doctors, whereas provision remains considerably more limited elsewhere. The objective of the proposed framework is therefore not to replace successful local programmes but to reduce unwarranted variation by providing a nationally coordinated structure that organisations can adapt to local circumstances. The phased implementation strategy proposed in this paper, including stakeholder engagement, faculty development, pilot implementation, quality assurance, and health economic evaluation, aims to ensure that future national adoption is evidence-based, practical, and sustainable.

This study has several limitations. The framework represents a conceptual, evidence-informed proposal based on published literature, national workforce evidence, and the authors' educational experience. It has not yet undergone formal stakeholder consultation, Delphi consensus methodology, or prospective pilot evaluation, nor has it received endorsement from national organisations. Although grounded in established educational theory and existing NHS infrastructure, its feasibility, acceptability, educational effectiveness, and cost-effectiveness remain to be demonstrated. This paper was not designed to compare educational outcomes between doctors in recognised postgraduate training programmes and those working outside them. Furthermore, important national outcome data remain unavailable, including the annual number of trauma and orthopaedic surgeons achieving specialist registration through the Portfolio Pathway, the average time required to complete the process, and the educational factors associated with successful progression. Finally, the non-deanery workforce is highly heterogeneous, encompassing locally employed doctors, speciality doctors, IMGs, MTI doctors, and clinicians pursuing the Portfolio Pathway, each with differing educational backgrounds, contractual arrangements, and career aspirations. Future refinement should therefore incorporate broad stakeholder engagement and evaluate these subgroups individually to determine whether specific educational interventions are required while maintaining consistent national standards.

Future directions

Future work should focus on validating and refining the proposed framework through national stakeholder consultation, formal consensus methodology, and prospective multicentre pilot implementation. Pilot programmes across representative NHS organisations should evaluate feasibility, acceptability, implementation fidelity, and educational effectiveness using objective measures, including workplace-based assessment completion, operative case diversity, portfolio evidence generation, FRCS examination outcomes, Portfolio Pathway application success, and workforce retention. Qualitative evaluation involving trainees, educational supervisors, and employing organisations should explore barriers and facilitators to implementation, while formal health economic evaluation should quantify implementation costs and determine cost-effectiveness. Future workforce research should also establish national outcome data describing progression through the Portfolio Pathway, including completion rates, time to specialist registration, and factors associated with successful progression. These findings would inform iterative refinement of the framework before wider regional or national implementation in collaboration with the British Orthopaedic Association, NHS England, the Joint Committee on Surgical Training, and other relevant stakeholders.

Ultimately, the National Non-deanery Training Initiative seeks to complement existing educational practice by promoting greater consistency, transparency, and equity in the development of doctors working outside recognised postgraduate training programmes. Rather than replacing successful local initiatives or altering existing routes to specialist registration, it provides a proposed national framework through which existing educational opportunities may be coordinated and strengthened. Although prospective validation remains essential, the framework represents an important first step towards improving educational support, strengthening workforce development, and contributing to the long-term sustainability of trauma and orthopaedic surgery within the NHS.

## Conclusions

The increasing contribution of non-deanery registrars, including international medical graduates, SAS doctors, and locally employed doctors, to the UK trauma and orthopaedic workforce necessitates a more structured and equitable approach to training and professional development. This technical report proposes a curriculum-aligned national framework that integrates educational supervision, workplace-based assessment, mentorship, portfolio development, operative competency monitoring, and protected training time within existing NHS structures. By converting routine clinical service into documented educational progression, the pathway offers a practical and scalable method of supporting progression towards consultant-level competence and specialist registration. Wider adoption of such a framework has the potential to improve educational consistency, enhance workforce retention, strengthen patient safety, and support the long-term sustainability of the trauma and orthopaedic workforce across the United Kingdom.
